# Inactivated Split MERS-CoV Antigen Prevents Lethal Middle East Respiratory Syndrome Coronavirus Infections in Mice

**DOI:** 10.3390/vaccines12040436

**Published:** 2024-04-18

**Authors:** Heejeong Seo, Yunyueng Jang, Dongmi Kwak

**Affiliations:** 1 PioneerVaccine, Inc., Chungnam National University, Daejeon 34134, Republic of Korea; hjseo2727@gmail.com; 2 College of Veterinary Medicine, Kyunpook National University, Daegu 41566, Republic of Korea

**Keywords:** MERS-CoV, inactivated split vaccine, coronavirus, prevention

## Abstract

Middle East respiratory syndrome coronavirus (MERS-CoV) causes fatal infections, with about 36% mortality in humans, and is endemic to the Middle East. MERS-CoV uses human dipeptidyl peptidase 4 (hDPP4) as a receptor for infection. Despite continued research efforts, no licensed vaccine is available for protection against this disease in humans. Therefore, this study sought to develop an inactivated fragmented MERS-CoV vaccine grown in Vero cells in an hDPP4-transgenic mouse model. Two-dose immunisation in mice with 15, 20, or 25 μg of spike proteins of inactivated split MERS-CoV antigens induced neutralising antibodies, with titres ranging from NT 80 to 1280. In addition, all immunised mice were completely protected, with no virus detection in tissues, weight loss, or mortality. The immunised splenocytes produced more cytokines that stimulate immune response (IFN-γ and TNF-α) than those that regulate it (IL-4 and IL-10). Taken together, the inactivated fragmented MERS-CoV vaccine is effective for the protection of mice against lethal MERS-CoV. Thus, the inactivated fragmented MERS-CoV vaccine warrants further testing in other hosts.

## 1. Introduction

Middle East respiratory syndrome coronavirus (MERS-CoV) was first found in an ill person in Saudi Arabia in 2012 [1]. As of January 2024, 2609 cases of MERS-CoV infection have been found in humans since 2012. Of these, 939 patients have died, suggesting a 35.9% mortality rate [2]. This zoonotic virus can infect camels and humans [3,4,5].

MERS-CoV clusters with the Coronaviridae family and has an RNA genome comprising 30,119 nucleotides [6,7,8]. Four structural proteins such as spike (S), envelope (E), membrane (M), and nucleocapsid (N) are present in the MERS-CoV genome [9,10,11]. The S protein exists on the MERS-CoV surface and is involved in host infection and pathogenesis [11]. In addition, this protein is used as a vaccine target [12,13,14].

MERS-CoV utilises dipeptidyl peptidase 4 (DPP4) as a receptor. It exists in the lower and upper respiratory tracts of humans and camels, respectively [15,16]. Moreover, camels are intermediate hosts for MERS-CoV infections in the Arabic Peninsula [17,18]. Camels infected with this virus show relatively mild clinical symptoms, making them difficult to identify [19].

Research on the development of safe and effective MERS-CoV vaccines to prevent human infection is underway, with a few MERS-CoV vaccines in Phase 1 clinical trials. A DNA vaccine (GLS-5300) encoding the MERS-CoV spike gene was used in a Phase 1 study at the Walter Reed Army Institute for Research Clinical Trials Center (Silver Spring, MD, USA) [20]. The ChAdOx1 MERS-CoV vaccine, which contains a replication-deficient simian adenovirus vector that expresses the MERS-CoV spike gene, was used in a Phase 1 clinical trial in the United Kingdom [21]. Similarly, a modified vaccinia virus Ankara vector vaccine expressing the MERS-CoV spike gene was used in a Phase 1 clinical trial in Germany [22].

Other methods for vaccine development, such as virus-like particles and live attenuated viruses, have been studied [23,24,25]. However, there remains no licensed MERS-CoV vaccine to date. Therefore, this study aimed to develop an inactivated split MERS-CoV vaccine using a human DPP4-transgenic mouse model.

## 2. Materials and Methods 

### 2.1. Animals, Cells, and Virus Culture

K18-hDPP4 mice encoding hDPP4 were provided by Dr. Paul B. McCray Jr. in Iowa, USA [26]. Mice had access to a chow diet and clean water at any time. A MERS-CoV (EMC2012-CA22°C) attenuated vaccine strain was developed through cold adaptation in Vero cells in our previous study [25], whereas Korean MERS-CoV/2015 was obtained from the Korean Centres for Disease Control and Prevention (KCDC). Vero-E6 cells purchased from the American Type Culture Collection (Manassas, VA, USA) were cultured in a humidified incubator (37 °C; 5% CO_2_) with minimal essential medium (MEM) (Merck, Burlington, MA, USA) and 10% foetal bovine serum (FBS) (Merck). The viruses were grown in Vero-E6 cells in a biosafety level 3 (BSL3) laboratory, which were approved by the KCDC. 

### 2.2. Production of Crude Extract of Inactivated MERS-CoV

Cultured Vero cells were inoculated with MERS-CoV (EMC2012-CA22°C) in MEM containing 1.5% bovine serum albumin (BSA; Merck) for 7 days in a humidified incubator (30 °C; 5% CO_2_). The supernatants of virus-infected cells were clarified through centrifugation at 7000× *g* for 10 min. Next, they were ultracentrifuged on a sucrose gradient of 25%/70% at 20,000× *g* for 3 h to purify the viruses. The purified viruses were inactivated using formaldehyde (0.02%) for 24 h at 4 °C. The viral titre prior to formaldehyde inactivation was about 1 × 10^12^ TCID50/mL. The formaldehyde was removed by multiple dialysis with PBS. The inactivated virus was then fragmented using Triton X-100 and Tween 80 (Merck) at 25 °C, and then, the detergent was removed using the column with Amberlite XAD-4 (Merck). After PBS dialysis, the final vaccine antigen was purified through a 0.2 μm syringe filter. 

### 2.3. Measurement of MERS-CoV Fragmented Protein Contents Using Single Radial Immunodiffusion

Inactivated fragmented MERS-CoV antigens were used for the vaccine study. First, the S protein was measured through single radial immunodiffusion using a standard MERS-CoV spike recombinant protein, which was expressed in a baculovirus expression system (Sino Biological, Beijing, China). Anti-rabbit hyperimmune serum produced in rabbits was subsequently immunised with the recombinant MERS-CoV spike protein (Sino Biological). A rabbit was immunised with 5 doses without an adjuvant (20 μg of spike protein per dose) at intervals of 4 weeks. 

A Petri dish was filled with 1% agar in PBS containing 730 μL of undiluted anti-rabbit MERS-CoV spike antibody, and circular wells (5 mm in diameter) were cut and discarded. A rabbit antibody was confirmed by using a standard S protein and single radial immunodiffusion. Next, a 2-fold series of a standard S protein and purified MERS-CoV split antigens were placed in separate wells on an agar plate. The reaction was incubated at 23 °C overnight and then stained with Coomassie Blue (Merck) before the ring diameters were measured. A standard curve was constructed using the ring diameters of the standards versus their concentrations. Finally, a standard curve was used to measure the spike concentration in the inactivated MERS-CoV split-virus vaccine. A known amount of MERS-CoV spike (1 mg/mL), which was 2-fold diluted in PBS, was run on the gel to measure ring diameters. Based on the ring-diameter standard curve of the recombinant S protein, the spike protein amount in the prepared vaccine antigens was about 1 mg/mL.

### 2.4. Efficacy of the Inactivated Fragmented MERS-CoV Vaccine in Mice

K18-hDPP4 mice (n = 13 per group) were intramuscularly (i.m) administered in their hind legs with three different doses of an inactivated fragmented mixture of the vaccine containing MERS-CoV spike proteins (15 μg, 20 μg, or 25 μg) in PBS. We used the vaccine antigen amount based on spike protein in the inactivated fragmented MERS-CoV vaccine since the neutralising antibody can be induced against the spike protein of MERS-CoV. We chose a starting dose of 15 μg in reference to the hemagglutinin amount (15 μg) of current inactivated influenza vaccine composition, since no inactivated MERS-CoV vaccine reference exists. They were subsequently boosted with the same doses of spike antigens four weeks later. Four weeks after this boost, the sera were bled before the immunised mice were intranasally (i.n.) challenged with 1 × 10^4^ plaque-forming units (pfu) of Korean MERS-CoV/2015. We used 1 × 10^4^ pfu to challenge mice since this dose caused 100% mouse mortality.

The infected mice were observed for changes in body weight and mortality. In addition, mice (n = 3 per group) were euthanised on day 6 post infection to determine the viral titres in the nasal turbinate, brain, and lungs and to stain lung tissues. As a control group, PBS mock-vaccinated mice were used. Day 6 was chosen to euthanise mice since mice started to show clear clinical signs such as a loss of body weight and ruffled hair at this point.

### 2.5. Measurement of Viral Neutralising Antibody Titres in the Immunised Sera

Sera (n = 10 per group) were obtained from the immunised K18-hDPP4 mice with 15 μg, 20 μg, or 25 μg MERS-CoV spike proteins at 8 weeks after the immunisation. They were then 10-fold diluted before being serially diluted 2-fold. Each 100 μL of diluted serum and 10^2^ TCID_50_/mL of Korean MERS-CoV/2015 (100 μL) were mixed in a 96-well plate. We used 100 μL of diluted serum and virus by considering the reaction volume of the well of a 96-well plate. Next, they were reacted for 1 h in an incubator (37 °C). The reaction mixture was placed to confluent Vero cells in a 96-well cell culture plate in a humidified incubator (5% CO_2_; 35 °C) before they were incubated for four days to observe cytopathic effects (CPE). Viral neutralising antibody titres were expressed as the highest diluted sera, where viral infection was inhibited in 100% of the cells. All reactions were performed four times.

### 2.6. Determination of Viral Titres in Tissue Samples

Homogenised tissue samples (0.1 g per tissue) were diluted in 1 mL of PBS (pH 7.4) before they were serially 10-fold diluted in MEM containing 1.5% BSA and used to infect confluent Vero cells in 96-well plates. The infected cells were incubated in a humidified incubator (5% CO_2_; 35 °C) for five days and subsequently observed under a microscope to determine CPE. Viral titres were calculated as log_10_TCID_50_/g based on the observed CPE, as previously described [27].

### 2.7. Pathological Tissue Staining

Lung tissues were fixed in 10% neutral buffered formalin before they were embedded in paraffin and cut into 5 μm pieces. The cut lung tissues were stained with haematoxylin (H) and eosin (E) (Merck) for 90 s. The analysis was carried out with an Olympus DP70 microscope (Olympus Corporation, Tokyo, Japan). Lung tissue pathologic scores were recorded based on the following criteria: 0, no clear sign; (1), mild inflammatory exudate and area of patchy edema with some disordered structure; (2), moderate inflammatory exudate and area of moderate alveolar thickening (<50%); (3), moderate–severe inflammatory exudate and severe alveolar thickening (>50%). 

### 2.8. Measurement of Cytokines in Splenocytes of the Immunised Mice

Splenocytes (2 × 10^6^/mL) from the mice (n = 5) immunised with two doses of 15 μg MERS-CoV spike proteins 8 weeks ago were stimulated with MERS-CoV spike proteins (2 μg/mL) in RPMI 1640 medium (Merck) containing 10% FBS in an incubator (5% CO_2_; 37 °C) for 24 h. We used the mixture of the viral fragment that contained 15 μg MERS-CoV spike proteins to immunise mice since the lowest dose (15 μg) completely protected the immunised mice against the lethal infections of MERS-CoV. The supernatant was subsequently collected. Next, T helper 1 (Th1) (TNF-α and IFN-γ) and T helper 2 (Th2) (IL-4 and IL-10) cytokines were measured using each ELISA kit (ThermoFisher Scientific, Waltham, MA, USA) following the manufacturer’s instructions. Briefly, 50 μL of splenocyte supernatant or standard was placed in wells coated with cytokine antibodies and reacted for 2 h at 23 °C. The biotin-labelled secondary antibody was added. The wells were then washed with 200 μL of wash buffer, to which 100 μL of Streptavidin–HRP was added before 1 h of incubation at 23 °C. Next, the wells in the plate were washed with the wash buffer in the kit. Then, 100 μL of 3,3′,5,5′-tetramethylbenzidine (TMB) was added and reacted for 30 min at 23 °C. A stop solution (100 μL) was added to stop the reaction before absorbance was recoded at 450 nm by a spectrophotometer (Bio-Rad, Hercules, CA, USA). The cytokine levels in the supernatant were measured using a standard curve.

### 2.9. Statistical Analysis

One-way ANOVA (Version 20.0; IBM Corp., Armonk, NY, USA) was used for statistical analysis. *p* < 0.05 was considered statistically significant. 

## 3. Results

### 3.1. Induction of Neutralising Antibodies in the Immunised Mice

**Figure 1:**
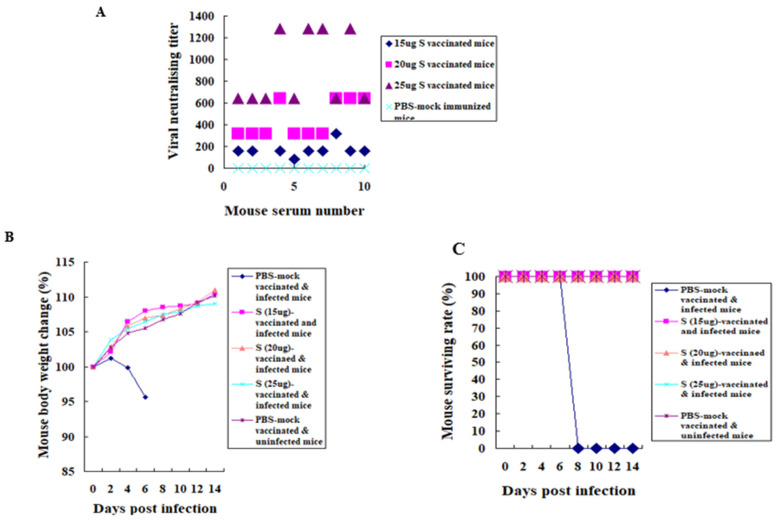
Antibody response in the immunised mice and protective efficacy of the immunised and challenged mice. Sera from K18-hDPP4 mice (n = 10 per group) i.m. immunised with two doses of 15 μg, 20 μg, or 25 μg of MERS-CoV spike proteins were used for measuring neutralising antibodies with Vero cells and Korean MERS-CoV/2015. Sera were 10-fold diluted in PBS and then 2-fold diluted in PBS (**A**). *p* < 0.05. The immunised mice were i.n. challenged with Korean MERS-CoV/2015 (1 × 10^4^ pfu). The inoculated mice were observed for body weight change (**B**) and mortality (**C**) for 14 days.

K18hDPP4 mice (n = 10 in each group) were i.m. immunised with two doses of 15, 20, or 25 μg of spike protein of the inactivated fragmented MERS-CoV vaccine. Sera were collected from the immunised mice and used to determine the titres of neutralising antibodies in Vero cells with the Korean MERS-CoV/2015. Overall, strong neutralising antibodies were activated in immunised mice (Figure 1). The induced ranges of neutralising antibody titres in mice immunised with 15, 20, or 25 μg of spike protein of the inactivated fragmented MERS-CoV vaccine antigen containing MERS-CoV structural proteins were 80–320 (Figure 1A), 320–640 (Figure 1A), and 640–1280 (Figure 1A), respectively. Sera from PBS-mock immunised mice were used as a control and no neutralising antibody was detected in them. Antibody titres suggest that the inactivated fragmented MERS-CoV antigens are immunogenic in mice.

### 3.2. Protection of the Vaccinated Mice

The immunised K18hDPP4 mice (n = 10 in each group) were i.n. infected with 1 × 10^4^ pfu of Korean MERS-CoV/2015. Challenged mice showed changes in body weight and mortality (Figure 1B,C). The mice immunised with two doses of 15, 20, or 25 μg of spike protein of the inactivated split MERS-CoV vaccine, as well as PBS-mock-vaccinated and uninfected mice, gained weight. In contrast, PBS-mock-vaccinated and infected mice lost body weight by up to 95.6% on day 6 post challenge (Figure 1B). All mice immunised with two doses of 15, 20, or 25 μg of spike protein of the inactivated split MERS-CoV vaccine survived. All PBS-mock-vaccinated and infected mice died before eight days post infection (Figure 1C). The result suggests that two doses of the inactivated MERS-CoV antigens can protect mice from the lethal infections of MERS-CoV. 

### 3.3. Tissue Viral Titres in Immunised and Challenged Mice

**Figure 2:**
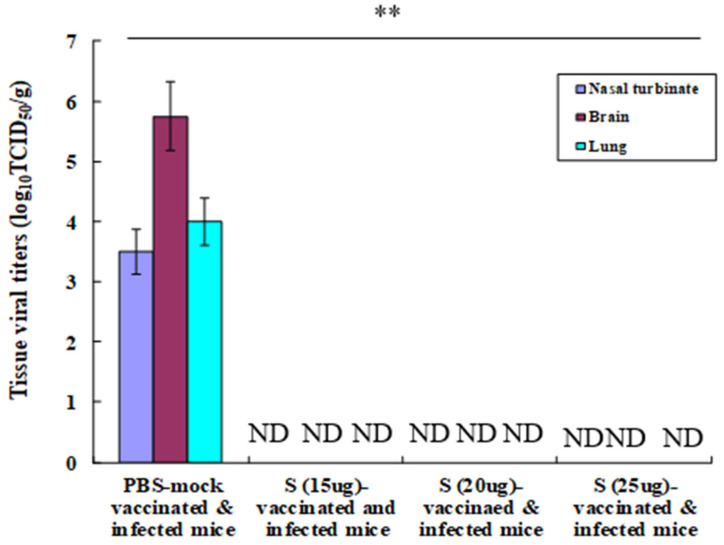
Tissue viral titres in the challenged mice. The immunised and infected K18-hDPP4 mice (n = 3 in each group) were euthanised on the sixth day post infection to detect viral titres in the nasal turbinate, brain, and lungs in Vero cells by log_10_TCID_50_/g. ND: undetected. Statistical analysis was performed with data from PBS-mock-vaccinated and infected mice and vaccinated and infected mice. ** *p* < 0.001.

The virus was not detected in the nasal turbinate, brain, and lung tissues of challenged mice that were immunised with the inactivated split MERS-CoV vaccine. Contrastingly, high viral titres were reported in the nasal turbinate (3.5 TCID50/g), brain (5.75 TCID50/g), and lung (4.0 TCID50/g) tissues of PBS-mock-infected mice (Figure 2). The result of viral titres in the tissues suggests that the inactivated fragmented MERS-CoV vaccine can provide sterile immunity to mice against the lethal MERS-CoV infections.

### 3.4. Lung Pathology in the Immunised and Challenged Mice

The haematoxylin and eosin staining results of the lung tissues collected from immunised and challenged K18-hDPP4 mice on day 6 post infection are shown in Figure S1. No interstitial pneumonia was observed in the lungs of infected mice inoculated with 15 (Figure S1C), 20 (Figure S1D), or 25 (Figure S1E) μg of spike protein of inactivated split MERS-CoV. No haemorrhage and neutrophil infiltration occurred in the lungs of the immunised mice. The same result was observed in PBS-mock-inoculated and uninfected mice (Figure S1A). In contrast, interstitial pneumonia with lymphocyte infiltration was observed in the lung tissues of PBS-mock-inoculated and infected mice (Figure S1B). Lung tissue clinical scores were recorded. No lung tissue pathology score was recorded for the lung tissues of the immunised and challenged mice or for the PBS-mock-immunised and uninfected mice, while the lung tissue pathology score for the PBS-mock-vaccinated and infected mice was 2.3 (Figure S2). The data on lung tissue pathology suggest that the inactivated fragmented MERS-CoV vaccine is effective for the prevention of pneumonia in mice against the lethal MERS-CoV infections.

### 3.5. Induction of Th1- and Th2-Type Cytokines in Splenocytes of Immunised Mice

In this study, both the immune stimulatory Th1 and the immune regulatory Th2 cytokines were induced. However, more Th1 cytokines were induced than Th2 cytokines (Figure S3). The amounts of the representative immune stimulatory TNF-α and IFN-γ induced were 47 pg/mL and 35 pg/mL, whereas those of the representative immune regulatory IL-4 and IL-10 induced were 15 pg/mL and 20 pg/mL, respectively. We used the spleen to collect cells since the spleen is one of the most important organs involved in the immune responses. The cytokine detection limit is 2 pg/mL. Cytokine data suggest that the inactivated fragmented MERS-CoV vaccine can induce more immune stimulatory cytokines than the immune regulatory cytokines in mouse T lymphocytes.

## 4. Discussion

MERS-CoV is endemic to camels in the Arabian Peninsula. However, sporadic transmission from infected camels to humans has been observed in recent years. This necessitates an effective and safe vaccine to protect humans from MERS-CoV infection. Our study developed an inactivated fragmented MERS-CoV strain in a mouse model. Our vaccine was immunogenic and effective in hDPP4-transgenic mice.

Immunisation of hDPP4-transgenic mice with two doses (15, 20, or 25 μg) of the spike protein of inactivated fragmented MERS-CoV antigens without an adjuvant induced a strong neutralising antibody. Here, all immunised mice were protected from lethal MERS-CoV infections without a loss of body weight or mortality. The efficacy of this vaccine was comparable to that shown in other MERS-CoV vaccine studies in mice [28,29,30]. The MERS-CoV spike receptor-binding domain-containing mRNA vaccine induced broad and durable neutralising antibodies and prevented mice from lethal infections [28]. Similarly, a single intranasal or intramuscular dose of a simian adenovirus (ChADOx1) vaccine expressing the full MERS-CoV spike protein protected mice against the lethal MERS-CoV challenge [29]. Moreover, recombinant parainfluenza virus 5 expressing the MERS-CoV spike protein-expressing parainfluenza virus 5 vector protected mice from MERS-CoV lethality by inducing neutralising antibodies and robust T cell stimulation [30].

In the current study, mice vaccinated with two doses (15, 20, or 25 μg) of spike proteins derived from inactivated fragmented MERS-CoV antigens prevented mice from MERS-CoV infection. After infection with MERS-CoV, the lungs of vaccinated mice did not show inflammatory lymphocyte infiltration. Contrary to our findings, an inactivated MERS-CoV vaccine in mice led to lung immunopathology with lymphocyte infiltration and reduced viral infection in a previous study [31]. Our vaccine induced strong neutralising antibodies, with neutralising titres ranging from 80 to 1280 in mice immunised with a spike protein (15, 20, or 25 μg) derived from inactivated fragmented MERS-CoV. The reason why inactivated fragmented MERS-CoV is immunogenic warrants further study. This vaccine completely inhibited viral infection, resulting in no lung immunopathology. In contrast, a previous study used a whole inactivated virus (100 μL of 1.2 × 10^8^ TCID50/mL) to immunise mice. However, the induced neutralising antibody titre was low (3.5–6.0), which led to lung immunopathology with reduced viral replication [31]. This indicates that sufficient antigens that induce strong neutralising antibodies against MERS-CoV are important for preventing lung immunopathology in MERS-CoV-infected mice.

We measured neutralising antibody titres to understand vaccine efficacy. For further study, the detection of total antibody titres in the immunised mice by ELISA may be useful to understand the immunogenicity of the inactivated fragmented MERS-CoV vaccine.

Single radial immunodiffusion was used to measure the spike amount in inactivated fragmented MERS-CoV vaccine antigens. Single radial immunodiffusion assay is one of the standard assays to measure hemagglutinin proteins in inactivated influenza vaccines, which were grown in eggs and cell culture [32]. Therefore, we used this assay to measure the spike antigens of our inactivated fragmented MERS-CoV antigens. Other methods such as SDS-PAGE may be also useful for the measurement of spike proteins in the vaccine antigens. 

We measured Th1 and Th2 cytokine induction in splenocytes from the immunised mice. Th1 cytokines (IFN-γ and TNF-α) were induced more than Th2 cytokines (IL-4 and IL-10). Further study may be needed to compare cytokine inductions between immunised and non-immunised mice. In addition, the study of cytokine inductions using proximal lymph nodes warrants further study.

## 5. Conclusions

This study aimed to develop a MERS-CoV vaccine using an hDPP4-transgenic mouse model. Human DPP4-transgenic mice were immunised with two doses (15, 20, or 25 μg) of a spike protein derived from inactivated split MERS-CoV antigens without an adjuvant. This vaccine generated strong neutralising antibodies. Thus, the inactivated split MERS-CoV vaccine was immunogenic in mice. Mice vaccinated with this antigen were completely protected from fatal MERS-CoV infection without a loss of body weight, death, viral replication in tissues, or pneumonia.

## Data Availability

The data are available upon request.

## Supplementary Materials

The following supporting information can be downloaded at: https://www.mdpi.com/article/10.3390/vaccines12040436/s1.
