# Supplementary material for: Inactivated Split MERS-CoV Antigen Prevents Lethal Middle East Respiratory Syndrome Coronavirus Infections in Mice
Source: Vaccines (Basel). 2024 Apr 18;12(4):436. doi: 10.3390/vaccines12040436 (PMC11053775; doi:10.3390/vaccines12040436)
Supplement: Supplementary file 1 [file vaccines-12-00436-s001.zip › vaccines-2939607-supplementary.pdf]

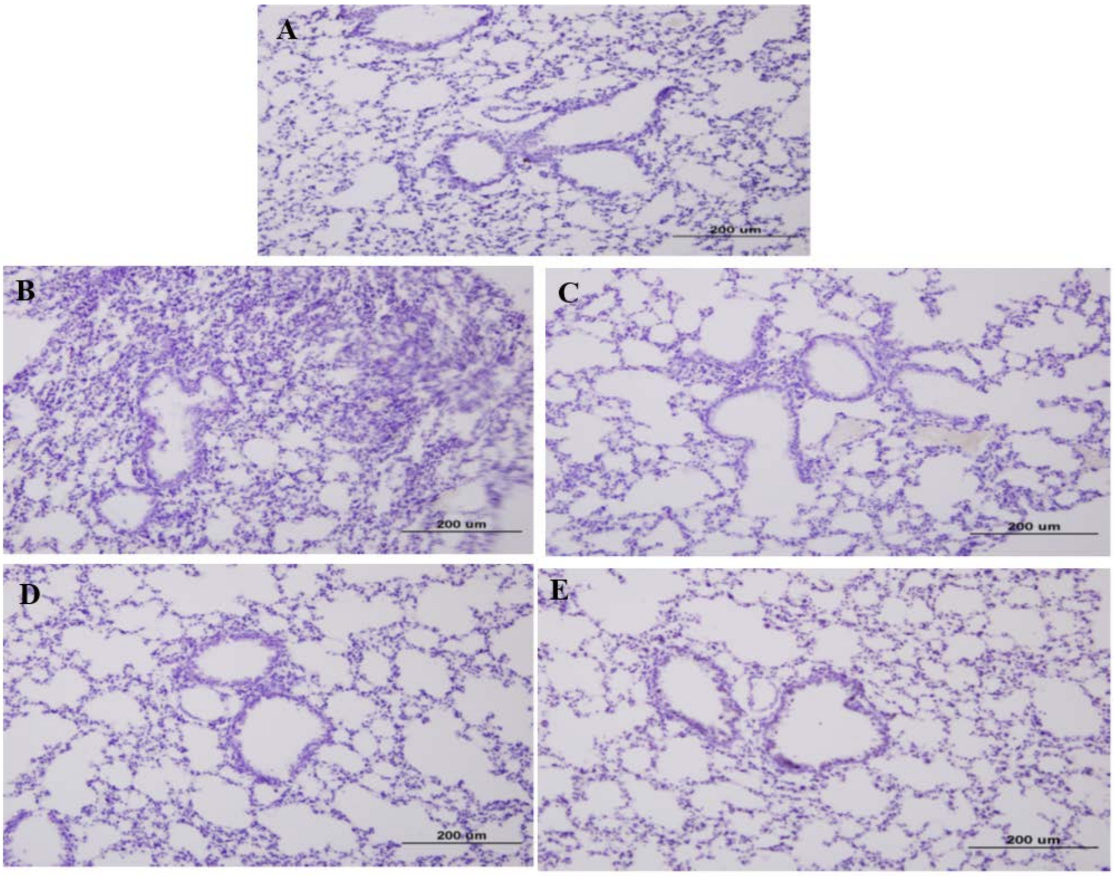

Figure S1. Lung pathology in the challenged mice. Lung tissues from Figure 2 were stained with H&E (X200) to observe tissue pathology in the immunized and challenged mice.

A, Lung tissue from PBS-mock inoculated and uninfected mouse; **B,** Lung tissue from PBS-mock inoculated and infected mouse; **C,** Lung tissue from the challenged mouse which were inoculated with 15μg spike protein; **D,** Lung tissue from the challenged mouse which were inoculated with 20μg spike protein; **E**, Lung tissue from the challenged mouse which were inoculated with 25μg spike protein.

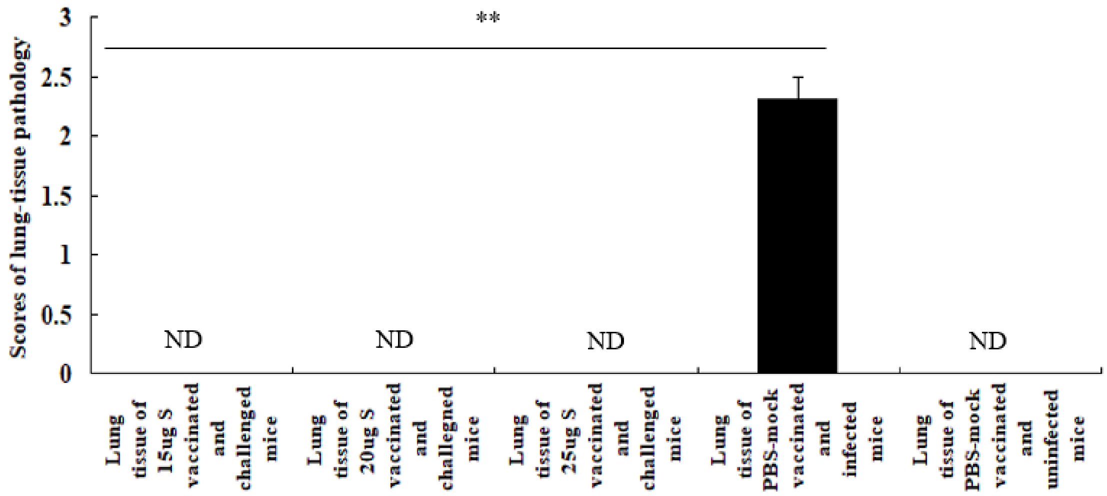

Figure S2. Lung clinical scores of Figure S1. Lung-tissue clinical scores were measured on the basis of the criteria: 0, no clear sign; 1, mild inflammatory exudate and area of patchy edema with some disordered structure; 2, moderate inflammatory exudate and area of moderate alveolar thickening (<50%); 3, moderate-severe inflammatory exudate and severe alveolar thickening (>50%). ND: None-detected. Statistical analysis was performed on data between lung tissue of PBS-mock vaccinated and infected mice and lung-tissue of the immunized and challenged mice. ** P<0.01.

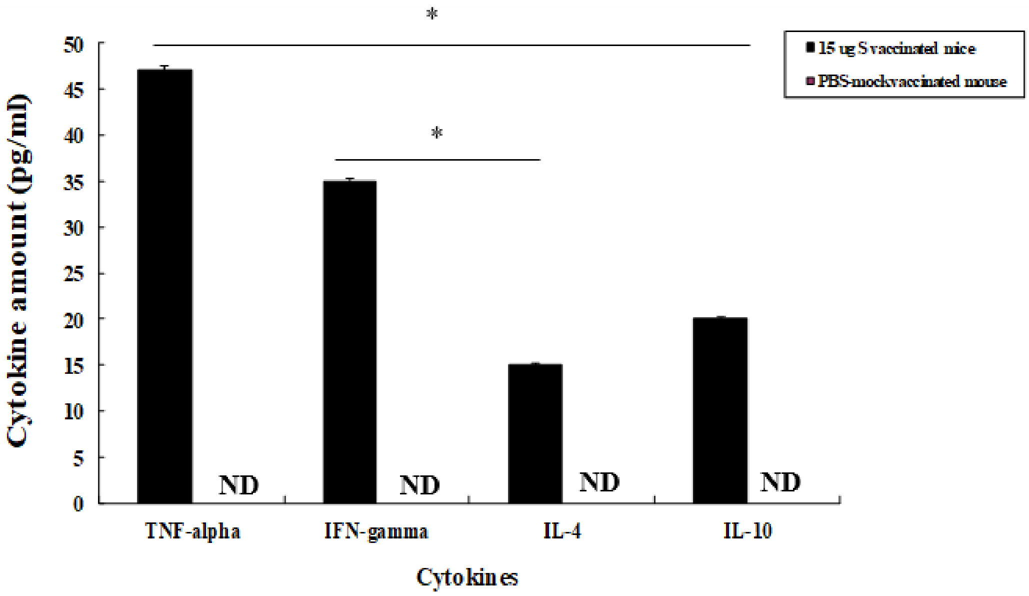

Figure S3. Cytokine profile in splenocytes in the immunized mice.

The supernatants from the stimulated splenocytes from the inoculated mice (n=5) with two doses of 15μg spike protein vaccines were used to measure immune stimulatory Th1 cytokines (TNF-α, IFN-γ) and immune regulatory Th2 cytokines (IL-4, IL-10) by ELISA. ND: Non-detected. Statistical analysis was performed data between Th1 cytokines and Th2 cytokines. *P<0.05
